# *Treponema pallidum* Detection at Asymptomatic Oral, Anal, and Vaginal Sites in Adults Reporting Sexual Contact with Persons with Syphilis

**DOI:** 10.3201/eid2910.230660

**Published:** 2023-10

**Authors:** Ei T. Aung, Christopher K. Fairley, Deborah A. Williamson, Francesca Azzato, Janet M. Towns, Rebecca Wigan, Eric P.F. Chow, Marcus Y. Chen

**Affiliations:** Melbourne Sexual Health Centre, Alfred Health, Melbourne, Victoria, Australia (E.T. Aung, C.K. Fairley, J.M. Towns, R. Wigan, E.P.F. Chow, M.Y. Chen);; Central Clinical School, Monash University, Melbourne (E.T. Aung, C.K. Fairley, J.M. Towns, E.P.F. Chow, M.Y. Chen);; The Doherty Institute for Infection and Immunity, Melbourne (D.A. Williamson);; Victorian Infectious Diseases Reference Laboratory, Melbourne (D.A. Williamson, F. Azzato);; Melbourne School of Population and Global Health, The University of Melbourne, Melbourne (E.P.F. Chow)

**Keywords:** syphilis, *Treponema pallidum*, sexually transmitted infections, PCR, polymerase chain reaction tests, syphilis screening, nucleic acid amplification tests, NAAT, sexual contacts, staging of syphilis, Australia

## Abstract

We investigated *Treponema pallidum* PCR positivity at mucosal sites (oral, anal, and vaginal sites) among adults who had sexual contact with a person with syphilis (syphilis contacts). All syphilis contacts had oral rinse and swab samples collected for testing. Men who have sex with men had anal swab and women had vaginal swab samples collected for testing, regardless of the presence of lesions. Of 407 persons tested, 42 (10%) had early syphilis diagnosed; of those, 19 (45%) tested positive by PCR from any anatomic site and had a positive serologic test. *T. pallidum* was positive from vaginal samples in 3 women, anal samples in 3 men, and oral cavity samples in 2 women and 3 men, without symptoms at those sites. Three women had no prior syphilis serologic test. *T. pallidum* detection at asymptomatic mucosal sites suggests early syphilis infections, particularly in cases that would conventionally be staged as latent syphilis of unknown duration.

Syphilis, caused by *Treponema pallidum*, results in substantial disease and death if left untreated. The World Health Organization (WHO) estimated a global burden of 6 million new syphilis infections in 2018, and syphilis remains a major public health challenge (*1*). High incidence of syphilis continues to persist among men who have sex with men (MSM) in high-income countries (*2*,*3*). Increases in syphilis among heterosexual populations and congenital syphilis have also been reported in many countries, including Australia (*4*). Early detection and treatment are essential in reducing the infectious period and transmission. Developing interventions aimed at improving syphilis control, including methods that can detect syphilis infection as early as possible, is essential. 

To identify early syphilis infection, persons who are at risk for syphilis infection should undergo screening. The conventional method for syphilis screening involves serologic testing, which consists of detecting *T. pallidum* antibodies by using *T. pallidum*–specific and nonspecific tests (*5*,*6*). However, a challenge with this method is the window period between the infection onset and the appearance of antibodies in very early syphilis, which can lead to a negative serologic result during that period, causing the infection to go undetected. Moreover, the sensitivity and specificity of *T. pallidum*–specific and –nonspecific serologic tests for syphilis vary by stages of infection (*7*,*8*). For example, *T. pallidum*–nonspecific tests are less sensitive in detecting primary syphilis (62%–78%) than in detecting secondary syphilis (97%–100%) (*7*). *T. pallidum*–specific tests such as immunoassays have a wide range of sensitivities for detecting primary syphilis (78%–96%), varying according to the specific immunoassay used (*9*)*.* Furthermore, those *T. pallidum*–specific immunoassays demonstrate persistent presence of treponemal antibodies in patients previously treated for syphilis (*9*), which can sometimes pose challenges in identifying a very early new syphilis infection when treponemal antibodies are present and *T. pallidum*–nonspecific tests are nonreactive. Therefore, laboratory methods that can enhance detection of very early syphilis infections are needed. 

Nucleic acid amplification tests (NAATs) such as PCR for *T. pallidum* have been shown to be highly sensitive for the detection of primary syphilis lesions (*10*–*14*), showing sensitivity ranging from 80% to 95% (*13*–*17*). Recent studies have shown *T. pallidum* is detectable by PCR not only from primary syphilis lesions at genital sites but also from other sites and sample types, including the anal canal, oral cavity, saliva, and urine (*14*,*18*–*23*). A study from the Netherlands detected *T. pallidum* by PCR in various mucosal tissues and body fluids in the early stages of syphilis, even in the absence of lesions (*20*). Those findings indicate that PCR can detect *T. pallidum* in the early stages of syphilis, with or without lesions at various mucosal sites. Consequently, PCR may be useful in detecting syphilis in asymptomatic persons at high risk before seroconversion takes place.

In this study, we undertook PCR testing for *T. pallidum* from adults who reported sexual contact with a person with syphilis by using oral, vaginal, and anal samples, even when symptoms and signs of syphilis were not reported from those sites. We hypothesized that some of those persons would have very early syphilis infection and *T. pallidum* would be detectable from those sites in the absence of lesions. We sought to determine whether PCR detection at those locations might precede the appearance of syphilis antibodies on serologic testing.

## Methods

In this cross-sectional study, we included men and women who reported sexual contact with a person with syphilis infection (hereafter, syphilis contacts), provided consent to having PCR testing for syphilis, and visited the Melbourne Sexual Health Centre (MSHC) during November 2018–March 2020. The study was approved by the Alfred Hospital Ethics Committee, Melbourne, Australia (project no. 474/18). MSHC is a public sexual health and HIV clinic in the state of Victoria, Australia, and provides ≈50,000 consultations/year. The clinic has an electronic medical record system that stores demographic and epidemiologic data.

Clients seeking care at the clinics were evaluated by a nurse after their registration. The nurse typically collects a brief account of the clients’ current condition and adds this information to their medical records before assigning them to healthcare professionals. Persons who had been in sexual contact with someone diagnosed with syphilis were identified on the basis of their own report. Those persons were notified about their exposure through either anonymous text messages or direct communication from the persons who had been in contact with them.

Contacts of syphilis were provided with a plain-language participant information sheet explaining PCR testing from mucosal sites. Verbal consent was obtained from those who agreed to have the *T. pallidum* PCR tests from the mucosal sites. The clinicians collected oral swab samples (all participants), anal swab samples (MSM only), and vaginal swab samples (women only) by using the Universal Transport Medium swab (Copan Italia, https://www.copangroup.com) for *T. pallidum* PCR testing. Participants also self-collected an oral rinse by gargling 10 mL of sterile water for *T. pallidum* PCR (i.e., mucosal screening PCR tests). *T. pallidum* PCR testing also was performed on swab samples taken from any syphilis lesions present (i.e., lesion PCR tests).

Participants who reported having symptoms suggestive of syphilis, such as anogenital or oral lesions, were examined by the clinician at the respective sites, and swab samples were taken from any lesion for *T. pallidum* PCR testing. Vaginal examinations were performed by using speculum if a woman reported any genital lesions or symptoms, and anoscopic examination was performed where a participant reported an anorectal lesion. Whether or not a participant required vaginal speculum or anoscopic examination was a decision made by the clinician on clinical grounds. We collected data on clinical examination findings retrospectively.

All participants had syphilis serologic testing performed and were offered syphilis treatment with intramuscular benzathine penicillin (2.4 mU single dose) or doxycycline (100 mg 2×/d for 2 weeks) for those with penicillin allergy. The participants were offered routine chlamydia and gonorrhea testing according to sexual transmitted infection (STI) testing guidelines depending on their sexual risk (*25**,**26*)*.* If persons were diagnosed with syphilis infection and considered to have latent syphilis of unknown duration, they were asked to return for further treatment with a total of 3 doses of weekly benzathine penicillin (or 4 weeks of doxycycline for those with penicillin allergy).

We defined MSM as men who have sex with men or with transwomen and bisexual as either men or women who had sex with both men and women (including transgender persons). We defined heterosexual as either men or women who had sex only with the opposite sex. We defined sexuality on the basis of self-reported sexual practice in the last 12 months and not on sexual identity.

E.T.A. reviewed medical records to capture information on types of partners (regular vs. casual), type of sex (anal, vaginal, or oral sex), condom use (condomless vs. with condoms), and signs and symptoms of syphilis at consultation. The information on types of partners was self-reported, and no formal definition of regular and casual partners was used in the study. Some participants might have defined regular partners as romantic partners (e.g., boyfriends or husbands) or casual sexual partners as regular contacts without romantic attachment (*24*).

### Laboratory Methods

We tested specimens at the Victorian Infectious Diseases Reference Laboratory (VIDRL; Melbourne, VIC, Australia), by using a TaqMan real-time PCR (ThermoFisher, https://www.thermofisher.com) targeting the *polA* gene of *T. pallidum* (*15*). We extracted DNA by using the Quick DNA/RNATM MagBead Extraction kit (Zymo Research, https://www.zymoresearch.com) on the Tecan Freedom EVO 100 automated system (Tecan, https://lifesciences.tecan.com). The PCR was designed at VIDRL by using the Primer Express software program (ThermoFisher, https://www.thermofisher.com), and the details of the *T. pallidum* PCR testing are described elsewhere (*15*). For a positive *T. pallidum* PCR sample, we reported a cycle threshold, which reflects the amount of nucleic acid in the sample.

We assessed serologic testing for syphilis by using a chemiluminescence immunoassay (CLIA) (DiaSorin, https://www.diasorin.com) and then confirmed results by using *T. pallidum* Particle Agglutination assay (TPPA) (https://www.fujirebio.com) and Rapid Plasma Reagin (RPR) (Becton Dickinson, https://www.bd.com). We performed routine screening for chlamydia and gonorrhea with NAAT by using Hologic Panther System Aptima Combo 2 assay (Hologic https://www.hologic.com) on the urine samples, vaginal swab samples, pharyngeal swab samples, and anal swab samples. We performed those screenings in line with MSHC testing guidelines and Australia’s STI guidelines (*25*,*26*). We obtained separate anal and oral swab samples for *T. pallidum* PCR and chlamydia and gonorrhea testing. We tested for herpes simplex virus from genital lesions by using an in-house TaqMan real-time PCR, targeting the glycoprotein B gene. We tested for *Mycoplasma genitalium* by using the ResistancePlus MG Assay (SpeeDx, https://plexpcr.com). We performed HIV screening by using the DiaSorin Liaison XL Murex HIV Ab/Ag chemiluminescence immunoassay (4th generation) and confirmed results by using Western blot.

### Identifying of Syphilis Cases

We identified all new syphilis cases by using laboratory classifications for early infectious syphilis and latent syphilis from the Australia Department of Health and the US Centers for Disease Control and Prevention (*27*,*28*). Of note, early latent syphilis in Australia is defined as syphilis infection acquired within the previous 24 months with no clinical evidence of syphilis (*28*). Staging of syphilis was undertaken by a senior sexual health physician, who determined the staging on the basis of the medical record and laboratory results, including results from external healthcare services.

### Statistical Analysis

We reported categorical variables as frequencies and percentages and continuous variables as medians and interquartile ranges (IQRs). We defined syphilis diagnosis as testing positive by *T. pallidum* PCR, serologic testing, or both. We calculated 95% CIs for syphilis diagnoses by using a binomial proportion CI. The study was stopped in March 2020 because of the COVID-19 pandemic. We performed all analyses by using Stata version 16 (StataCorp LLC, https://www.stata.com).

## Results

A total of 407 contacts had >1 specimen for PCR screening (oral and anal in MSM, oral in heterosexual men, and oral and vaginal in women). Of the 407 contacts, 339 were MSM, 22 were heterosexual men, 20 were heterosexual women, 1 was a bisexual woman, and 25 were bisexual men. Among the contacts living with HIV (16%, n = 67), most were MSM (94%, n = 63); one third of contacts (33%, n = 134) were using HIV preexposure prophylaxis. One fifth of the contacts (20%, n = 85) had a history of syphilis infection, and a small number (9%, n = 35) reported ever injecting drugs. The median age of the syphilis contacts was 32 years (IQR 27–40 years).

Nearly half (47%, n = 193) of the contacts reported having a casual partner as their syphilis contact, whereas 35% (n = 144) reported a regular partner as the contact. Most (71%, n = 290) contacts reported condomless sex during anal or vaginal sex, and 17% (n = 69) had signs or symptoms suggestive of syphilis (Table 1).

**Table 1:** Possible syphilis symptoms described by 69 patients in retrospective study of men and women who visited the Melbourne Sexual Health Centre, Melbourne, Victoria, Australia, during November 2018–March 2020*

Symptom	No. patients
Oral symptoms, n = 15	
Oral ulcers: mouth ulcers, gum ulcers, tongue ulcers†	11
Lip sores or ulcers	4
Anal symptoms, n = 18	
Anal or perianal ulcers/blisters/lesions, painful and painless	7
Anal lump	2
Anal or perianal rash, red rash	1
Anal pain: severe, discomfort, associated with mucous discharge	5
Tenesmus	2
Anal itch†	1
Penile or genital symptoms, n = 17	
Penile lesions/sores on glans penis, foreskin	8
Penile rash, including red spots, pimple-like	5
Penis lump	2
Scrotal lump and rash, flaky skin†	2
Nonorogenital symptoms, n = 18	
Body rash: torso, back, flank, palms, soles‡	12
Rash/sores on shin, leg, thigh	4
Lump on eyelid, conjunctivitis	2
Systemic symptoms, n = 21	
Headache	3
Fever, including night sweats, chills	6
Fatigue/lethargy	2
Influenza-like symptoms, unwell, sore throat	5
Blurred vision	2
Tinnitus	1
Swollen lymph nodes: groin, submental	2

*Some participants presented with >1 symptom, and therefore, the total would not add up to 69. †The participant had tongue ulcer, flaky skin on scrotum and itchy anus with PCR positive test results from tongue, scrotum, and anus. ‡Some descriptions include spots, itchy papules, and red patches.

A total of 42 contacts (10%, 95% CI 8%–14%) had syphilis infection diagnosed (case-patients) (Figure). Of those, 33 were MSM, 5 were women, 2 were heterosexual men, and 3 were bisexual men. Syphilis was detected by positive serologic testing alone in the absence of positive PCR in 21 cases (50%, 95% CI 34%–66%). Nineteen cases (45%, 95% CI 30%–61%) were detected by positive serologic tests together with positive *T. pallidum* PCR; 9 of those case-patients had *T. pallidum* detected by PCR from >1 mucosal sites (oral, anal, or vaginal) in the absence of lesions at these sites (Table 2). The remaining 10 of the 19 case-patients had signs and symptoms suggestive of syphilis and were *T. pallidum*–positive from mucosal sites, lesions sites, or both (Table 3). Of the 42 syphilis case-patients, 2 had *T. pallidum* PCR detected from penile lesions with a negative serologic test. Because those 2 case-patients had *T. pallidum* PCR detected only from lesion PCR tests, they are not discussed further.

**Figure:**
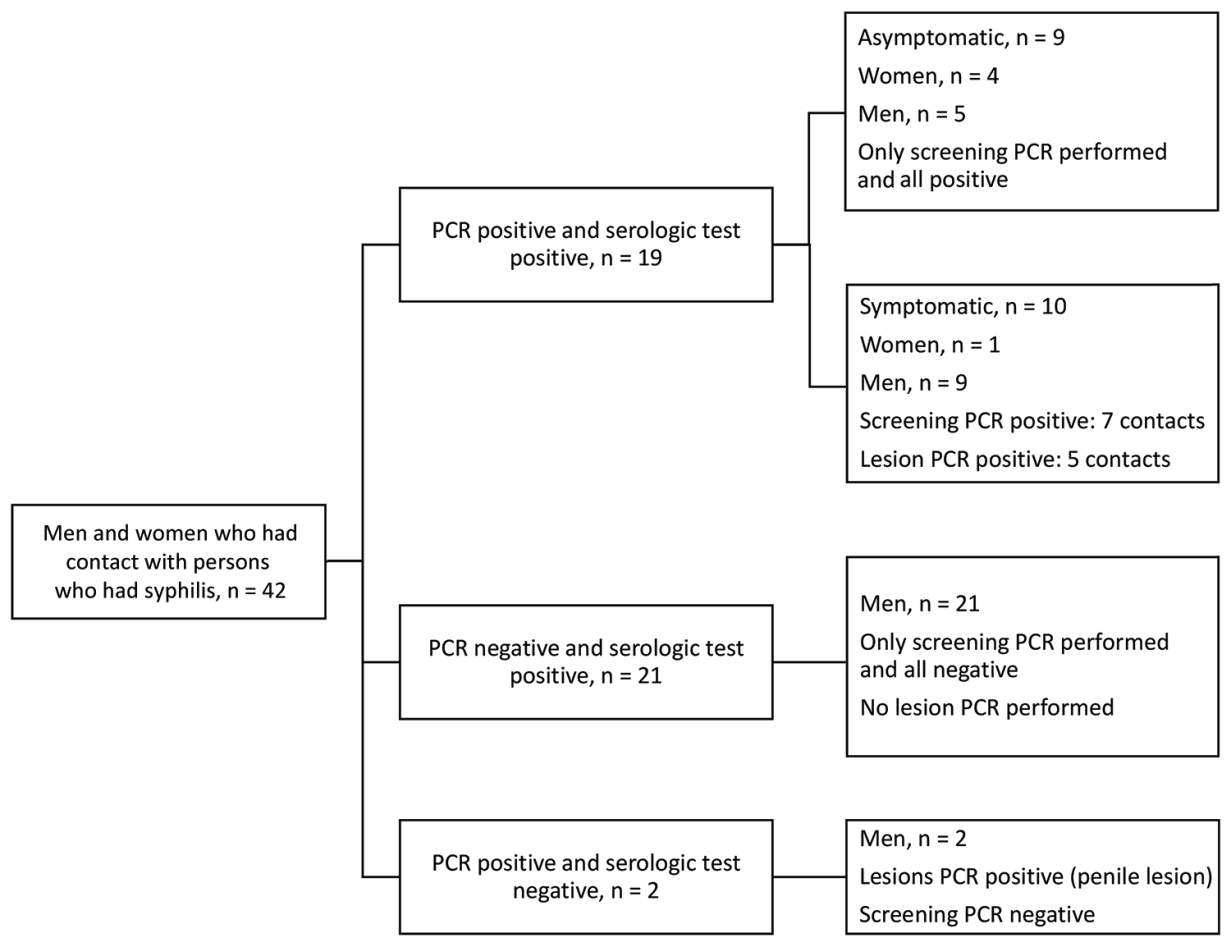
Flowchart of syphilis infections among men and women who had contact with persons who had syphilis, categorized by positive PCR and serologic test results and presence or absence of symptoms, in retrospective study of patients who visited the Melbourne Sexual Health Centre, Melbourne, Victoria, Australia, during November 2018–March 2020.

**Table 2:** Characteristics of 9 syphilis patients without signs or symptoms of syphilis who had PCR detection of *Treponema pallidum* from oral, anal, or vaginal sites in retrospective study of men and women who visited the Melbourne Sexual Health Centre, Melbourne, Victoria, Australia, during November 2018–March 2020*

Group by sexual practice	HIV/PrEP status	Signs on examination	Mucosal PCR sites (value if positive†)				Serologic test results		Previous serologic test results (RPR titer if reactive)	Staging of syphilis
			Oral cavity	Vagina	Anal		Current RPR titer	Previous syphilis serologic test		
Woman‡	Neg	No	–	+ (31)	ND		1:8	8 y	Negative	Primary
Woman§	Neg	ND	–	+ (36)	ND		1:2	None	NA	Primary
Woman‡	Neg	No	+ (33)	ND	ND		1:128¶	None	NA	Primary
Woman‡	Neg	ND	+ (32)	+ (37)	ND		1:16	None	NA	Early latent
Bisexual man	Neg	ND	–	NA	+ (35)		Nonreactive#	8 mo	Negative	Early latent
MSM	Neg	No	–	NA	+ (26)		1:32	12 mo	Negative	Early latent
MSM	HIV	No	+ (30)	NA	–		1:256¶	10 mo	+ (1:8)	Primary
MSM	HIV	No	+ (36)	NA	–		1:128¶	10 mo	Negative	Primary
MSM	PrEP	No	+ (36)	NA	+ (32)		1:32	2 mo	Nonreactive	Primary

*MSM, men who have sex with men; NA, not applicable; ND, not done; neg, HIV-negative and not taking PrEP; PrEP, preexposure prophylaxis for HIV; RPR, rapid plasma reagin; woman, heterosexual woman; –, negative by *T. pallidum* PCR; +, positive by *T. pallidum* PCR. †Cycle threshold value of *T. pallidum* PCR. ‡The regular partners (of >1 year duration) had early syphilis (primary and secondary) diagnosed. §The casual partner had secondary syphilis diagnosed. ¶These 3 patients had high RPR titers with oral PCR detection. We attribute oral PCR detection to shedding after dissemination of *T. pallidum* on the basis of the high RPR titer, although this finding could be attributable to occult lesions in the oral cavity. #Positive *T. pallidum*–specific antibodies and nonreactive RPR with negative *T. pallidum*–specific antibodies 8 months prior.

**Table 3:** Characteristics of 10 syphilis patients with signs and symptoms of syphilis who had a positive serologic test and *Treponema pallidum* PCR detection from mucosal sites or lesion sites in retrospective study of patients who visited the Melbourne Sexual Health Centre, Melbourne, Victoria, Australia, during November 2018–March 2020*

Group by sexual practice	HIV/PrEP status	Signs on examination	Mucosal PCR sites (value if positive†)				Serologic test results			Previous serologic test results (RPR titer if reactive)	Staging of syphilis
			Oral cavity	Vagina	Anus		Lesion PCR sites (value if positive†)	Current RPR titer	Previous syphilis serologic test		
Woman	Neg	Oral ulcers	+ (36)	–	–			1:64	24 mo	Negative	Primary
MSM	Neg	Tender anal ulcers	–	NA	+ (30)			1:16	3 wks	1:1	Primary
MSM	HIV	Torso and hand rash	+ (31)	NA	+ (30)			1:64	5 mo	Nonreactive	Secondary
MSM	PrEP	Torso rash, penis and perianal rash	+ (34)	NA	+ (30)			1:128	2 mo	Nonreactive	Secondary
MSM‡	Neg	Maculopapular rash on torso and soles	–	NA	+ (26)			1:256	12 mo	Unknown	Secondary
MSM‡	Neg	Ulcer on tongue, scrotal rash	+ (32)	NA	+ (36)		Tongue, + (30); scrotum, + (39)	1:32	26 mo	Unknown	Primary
MSM	HIV	Penile ulcer	+ (34)	NA	+ (40)		Penis, + (34)	1:128	4 mo	Negative	Primary
Bisexual man	Neg	Penile ulcers	–	NA	–		Penis, + (38)	1:32	15 mo	Negative	Primary
MSM	HIV	Penile ulcers, torso rash	–	NA	–		Penis, + (36)	1:64	12 mo	Negative	Secondary
MSM	HIV	Palms and feet rash, ulcers on penis, and nodules on scrotum	–	NA	–		Penis, + (36) scrotum, –; palms, –; soles, –	1:128	16 mo	1:1	Secondary

*MSM, men who have sex with men; NA, not applicable; ND, not done; neg, HIV negative and not taking PrEP; PrEP, preexposure prophylaxis for HIV; RPR, rapid plasma reagin; woman, heterosexual woman; –, negative by *T. pallidum* PCR; +, positive by *T. pallidum* PCR. †Cycle threshold value of *T. pallidum* PCR. ‡These 2 patients had syphilis treated at other clinics previously, but the exact RPR at that time is unknown.

### Syphilis Cases Detected by *T. pallidum* PCR and Serologic Testing

Among the 9 asymptomatic case-patients (i.e., PCR positive and no visible lesions), 4 were women, 4 were MSM, and 1 was a bisexual man. The median age was 27 years (IQR 24–31 years). *T. pallidum* PCR was detected from >1 of the mucosal screening PCR tests (58% [95% CI 37%–78%], 14/24): oral swab, oral rinse, anal swab, or vaginal swab (Table 2). The RPR titer ranged from nonreactive to 1:256 (median RPR 1:32, IQR 1:8–1:128).

Among the 10 symptomatic case-patients (i.e., PCR positive and visible lesions), 1 was a woman, 8 were MSM, and 1 was a bisexual man. The median age was 32 years (IQR 24–39 years). In this group, 69% (95% CI 52%–83%, 27/39) of *T. pallidum* PCR specimens were positive from the mucosal screening PCR tests, the lesion PCR tests, or both. The RPR titer ranged from 1:32 to 1:256 (median RPR 1:64, IQR 1:32–1:128) (Table 3). Of the 10 symptomatic case-patients, 7 had positive PCR from mucosal screening sites with or without positive PCR from lesion sites. Among these 7 case-patients, 5 had positive PCR only from mucosal screening sites; 3 had signs and symptoms of secondary syphilis. Two case-patients had positive PCR from both mucosal screening sites and lesion sites (Table 3).

### Syphilis Cases Detected by Positive Serologic Tests in the Absence of *T. pallidum* PCR Detection

Twenty-one case-patients had positive syphilis serologic tests but no positive *T. pallidum* screening results (lesion PCR was not performed) (Table 4). This group included 19 MSM, 1 heterosexual man, and 1 bisexual man. The median age in this group was 33 years (IQR 28–40 years). Most (86%, 18/21) were asymptomatic for syphilis except 3 case-patients. Two men with symptoms had secondary syphilis diagnosed and had a generalized body rash. One man with symptoms had a headache and blurred vision, consistent with symptoms of neurosyphilis, and a resolving body rash. *T. pallidum* lesion PCR tests were not performed on the 3 men with symptoms. The RPR titer ranged from nonreactive to 1:128 (median RPR 1:4, IQR nonreactive to 1:32).

**Table 4:** Characteristics of 21 syphilis patients with positive serologic tests in the absence of PCR detection from mucosal sites or lesion sites in retrospective study of patients who visited the Melbourne Sexual Health Centre, Melbourne, Victoria, Australia, during November 2018–March 2020*

Group by sexual practice	HIV/PrEP status	Signs and symptoms of syphilis	Current RPR titer	Previous syphilis serologic test	Previous serologic test result (RPR titer if reactive)	Staging of syphilis
MSM	Neg	No	1:4	8 mo	Nonreactive	Early latent
MSM†	PrEP	No	Nonreactive	18 mo	Nonreactive	Early latent
MSM	PrEP	No	Nonreactive	10 mo	Negative	Early latent
MSM	PrEP	Not examined	Nonreactive	3 mo	Negative	Early latent
MSM	Neg	No	1:16	5 mo	Nonreactive	Early latent
MSM	HIV	No	Nonreactive	6 mo	Negative	Early latent
MSM	PrEP	No	1:128	4 mo	Negative	Early latent
MSM	Neg	No	1:32	12 mo	Negative	Early latent
MSM	Neg	Not examined	1:64	4 mo	Negative	Early latent
MSM	HIV	Not examined	1:64	1 mo	Negative	Early latent
MSM	PrEP	Not examined	1:4	8 mo	1:1	Early latent
Heterosexual man	Neg	Not examined	1:8	8 mo	Negative	Early latent
Bisexual man	Neg	Macular rash on palms	1:32	None	None	Secondary
MSM	Neg	Rash/papules on penis, maculopapular rash on hands and back	1:32	14 mo	Nonreactive	Secondary
MSM	PrEP	Not examined	Nonreactive	11 mo	Negative	Early latent
MSM^§^	Neg	Blurred vision and headache, Resolved body rash before presentation	1:64	19 mo	Negative	Secondary/neurosyphilis
MSM	HIV	Not examined	1:4	3 mo	Nonreactive	Early latent
MSM	PrEP	No	Nonreactive	6 mo	Negative	Early latent
MSM^‡^	Neg	No	1:2	None	None	Late latent
MSM	PrEP	Not examined	Nonreactive	1 mo	Negative	Early latent
MSM	HIV	Not examined	1:32	2 mo	Nonreactive	Early latent

*MSM, men who have sex with men; neg, HIV negative and not taking PrEP; nonreactive, nonreactive RPR; PrEP, preexposure prophylaxis for HIV; RPR, rapid plasma reagin. †IgM was reactive on serologic test. ‡Treated for late latent syphilis. §Subsequently had neurosyphilis diagnosed.

### Positivity of *T. pallidum* PCR

The positivity of *T. pallidum* PCR from the mucosal screening tests (oral, anal, and vaginal sites) was 3% (95% CI 2%–5%; n = 24) from 776 specimens, whereas the positivity from the suspected syphilis lesions was 16% (95% CI 7%–30%; n = 8) from 50 specimens tested from various sites, such as the penis, perianal, scrotum, and tongue (Table 5). The concordance of oral rinse (2%, 8/387) and oral swab (3%, 11/352) PCR was 99% (n = 352/355).

**Table 5:** Patients testing positive on *Treponema pallidum* PCR at mucosal screening sites and lesion sites in retrospective study of patients who visited the Melbourne Sexual Health Centre, Melbourne, Victoria, Australia, during November 2018–March 2020

Sites tested by *T. pallidum* PCR	No. tested	No. (%) positive by *T. pallidum* PCR
Mucosal sites*	776	24 (3.0)
Oral cavity†	405	12 (3.0)
No oral symptoms	339	8 (2.4)
Oral symptoms present	66	4 (6.1)
Anal swab	352	9 (2.6)
No anal symptoms	291	4 (1.4)
Anal symptoms present	61	5 (8.2)
Vaginal swab	19	3 (13.6)
No vaginal symptoms	18	3 (16.7)
Vaginal symptoms present	1	0
Lesion sites‡	50	8 (16.0)
Penile swab	23	6 (26.0)
Other sites (perianal: 13)	27	2§ (7.4)

*Oral cavity, anus, and vagina. †Specimens tested from oral cavity were oral swab, oral rinse, or both. Oral swab was positive in 11/352 (3%) samples. Oral rinse was positive in 8/397 (2%) samples. ‡Penis, perianal, labia, mouth ulcer, palms, soles, pubic, right groin, scrotum, and tongue. §Positive PCR from specimens tested at tongue and scrotum.

### Presence or Absence of Signs and Symptoms of Syphilis

Among 338 asymptomatic contacts, 8% (27, 95% CI 5–11) had syphilis infection diagnosed on the basis of positive serologic tests with or without positive *T. pallidum* PCR results. Of the 338 contacts, 56% (180/338) received an examination. Among 69 contacts with signs and symptoms of syphilis, 22% (15, 95% CI 13–33) had syphilis infection diagnosed on the basis of positive serologic tests, positive *T. pallidum* PCR, or both.

### Co-infection with Other STIs

A total of 32 contacts (8%, 32/407) had >1 STI other than syphilis diagnosed. All of them were MSM, and among them, 6 had syphilis diagnosed. Five had syphilis diagnosed on positive PCR and serologic testing, whereas 1 had syphilis diagnosed on a positive serologic test alone. Among the 32 contacts, 17 were positive for chlamydia, and 12 contacts were positive for gonorrhea. Five contacts were positive for *Mycoplasma genitalium* infection*,* and 2 contacts tested positive for anal herpes simplex virus. Among the STIs other than syphilis, anorectal chlamydia was the most common infection (n = 15). 

## Discussion

In this study of men and women reporting sexual contact with a person with syphilis, we did not identify any persons with PCR detection of *T. pallidum* from oral, anal, or vaginal sites where serologic testing was negative, indicating that using PCR for screening of syphilis contacts at mucosal sites might not provide any additional benefit over existing syphilis screening using serologic testing. However, we found a proportion of men and women who tested positive for *T. pallidum* by PCR from the oral cavity, anus, or vagina in the absence of signs or symptoms of syphilis at those sites or elsewhere. In some cases, serologic testing for syphilis was positive in the absence of negative serologic testing within the previous 2 years. Those persons would conventionally be staged as having latent syphilis of unknown duration, and they probably were treated for possible late latent infection. However, detection of *T. pallidum* by PCR from oral, anal, or vaginal sites, in conjunction with the syphilis contact status, suggests that those infections probably were early asymptomatic infections.

The findings indicate that PCR-based testing for *T. pallidum* at mucosal sites may be useful in assisting with staging of syphilis infection. Consequently, this approach could provide guidance to clinicians and patients regarding treatment duration. Furthermore, *T. pallidum* PCR testing at mucosal sites has the potential to aid in partner notification, particularly in cases where an early infectious syphilis is diagnosed and the duration of infection is uncertain. *T. pallidum* PCR may complement current methods of staging, which rely on various factors, including the presence of signs of early syphilis, the patient’s history of syphilis and treatment, sexual history (including contact with a partner with syphilis infection), and past and current laboratory results (including serologic testing and direct detection methods using molecular assays).

We identified several asymptomatic women who had positive syphilis serologic testing and PCR detection of *T. pallidum* from the mucosal screening sites (the vagina, oral cavity, or both). Those women had never been serologically tested for syphilis before or had been tested >2 years previously. We also identified several asymptomatic MSM who had positive syphilis serologic testing and PCR detection of *T. pallidum* from the anus, oral cavity, or both. In contrast to the women, all those men had been serologically tested for syphilis within the previous 2 years. The difference in serologic testing between women and men reflects frequent serologic screening for syphilis being well established among MSM in Australia but less so among women in urban centers because syphilis has only emerged as a major public health concern among women in that setting in recent years.

Several studies have examined the role of NAAT in syphilis screening (*20*,*29*). A US study compared the use of transcription-mediated amplification (TMA) assay performed on rectal and pharyngeal mucosa in MSM with routine serologic testing and found 2 additional syphilis cases diagnosed on TMA testing before positive serologic testing (*29*). Patients in both cases had TMA detection in rectal swabs: 1 did not have symptoms, and the other had anal symptoms. In contrast, our study did not identify syphilis cases diagnosed on *T. pallidum* PCR from mucosal sites before positive serologic testing.

In our study, we found that 40% of the 42 confirmed early syphilis cases among men and women had PCR detection of *T. pallidum* from >1 of the mucosal sites (oral, anus, and vagina), with or without lesions at these sites. Of note, we show PCR detection of *T. pallidum* in asymptomatic women. *T. pallidum* detection by PCR in women has been shown in other studies but predominantly from genital lesions (*16*,*30*–*32*). The presence of *T. pallidum* at those mucosal sites may represent the site of inoculation (and hidden primary lesions) or dissemination from a distant site (*19*). Several other recent studies showed that *T. pallidum* can be detected from the oral cavity or anus of men and women with early syphilis infection (*18*–*23*). Detection of *T. pallidum* by PCR was reported in ≈24% of 200 MSM with confirmed early syphilis infection with or without lesions at the oral cavity and anus (*18*). Those studies suggest that syphilis probably is infectious from oral, anal, and vaginal sites in the absence of local signs and symptoms.

The first limitation of our study is that it was terminated prematurely because of the COVID-19 pandemic, which limited the sample size. The number of women in the study was small because the clinic population was predominantly male, and fewer women attended. The sexual partners reported by contacts might not have actually had syphilis, given that we were unable to confirm their diagnosis; if so, we may have overestimated syphilis infections in this study group. Not all contacts who self-reported that they were asymptomatic had examination of oral, vaginal, and anal sites by the clinician (46% did not undergo examination), which could have led to occult lesions at those sites being missed. The *T. pallidum* PCR we used might have lower sensitivity than other *T. pallidum* molecular assays, such as TMA, resulting in a lower number of PCR-positive mucosal screening tests (*29*,*33*). Further, current data are not sufficient to indicate that *T. pallidum* PCR positivity at a mucosal site is a proof of early syphilis infection. *T. pallidum* might reflect contamination from very recent sexual intercourse with a person with syphilis infection, such as residual semen at the mucosal site.

Overall, we conclude that *T. pallidum* PCR screening from mucosal sites (oral, anus, and vagina) may not have added benefit over screening using serologic testing. It may, however, have a role in assisting with syphilis staging, particularly in the absence of syphilis lesions. A positive PCR result from asymptomatic mucosal sites may help identify early infections in persons who would otherwise be classified as having latent syphilis of unknown duration. However, such an interpretation requires a correlation with additional sexual behavioral information and the broader clinical context. *T. pallidum* PCR screening at mucosal sites may be especially relevant in populations that do not undergo regular syphilis screening with serologic testing. 

## References

[1] World Health Organization. Report on global sexually transmitted infection surveillance, 2018. Geneva: World Health Organization; 2018.

[2] Tsuboi M, Evans J, Davies EP, Rowley J, Korenromp EL, Clayton T, et al. Prevalence of syphilis among men who have sex with men: a global systematic review and meta-analysis from 2000-20. Lancet Glob Health. 2021;9:e1110–8. 10.1016/S2214-109X(21)00221-7 34246332 PMC9150735

[3] Kojima N, Klausner JD. An update on the global epidemiology of syphilis. Curr Epidemiol Rep. 2018;5:24–38. 10.1007/s40471-018-0138-z 30116697 PMC6089383

[4] Aung ET, Chen MY, Fairley CK, Higgins N, Williamson DA, Tomnay JE, et al. Spatial and temporal epidemiology of infectious syphilis in Victoria, Australia, 2015–2018. Sex Transm Dis. 2021;48:e178–82. 10.1097/OLQ.0000000000001438 33859143

[5] Luo Y, Xie Y, Xiao Y. Laboratory diagnostic tools for syphilis: current status and future prospects. Front Cell Infect Microbiol. 2021;10:574806. 10.3389/fcimb.2020.574806 33628742 PMC7897658

[6] Peeling RW, Mabey D, Kamb ML, Chen X-S, Radolf JD, Benzaken AS. Syphilis. Nat Rev Dis Primers. 2017;3:17073. 10.1038/nrdp.2017.73 29022569 PMC5809176

[7] Tuddenham S, Katz SS, Ghanem KG. Syphilis laboratory guidelines: performance characteristics of nontreponemal antibody tests. Clin Infect Dis. 2020;71(Suppl 1):S21–42. 10.1093/cid/ciaa306 32578862 PMC7312285

[8] Park IU, Tran A, Pereira L, Fakile Y. Sensitivity and specificity of treponemal-specific tests for the diagnosis of syphilis. Clin Infect Dis. 2020;71(Suppl 1):S13–20. 10.1093/cid/ciaa349 32578866 PMC7312216

[9] Park IU, Fakile YF, Chow JM, Gustafson KJ, Jost H, Schapiro JM, et al. Performance of treponemal tests for the diagnosis of syphilis. Clin Infect Dis. 2019;68:913–8. 10.1093/cid/ciy558 29986091 PMC6326891

[10] Heymans R, van der Helm JJ, de Vries HJ, Fennema HS, Coutinho RA, Bruisten SM. Clinical value of *Treponema pallidum* real-time PCR for diagnosis of syphilis. J Clin Microbiol. 2010;48:497–502. 10.1128/JCM.00720-09 20007388 PMC2815629

[11] Gayet-Ageron A, Lautenschlager S, Ninet B, Perneger TV, Combescure C. Sensitivity, specificity and likelihood ratios of PCR in the diagnosis of syphilis: a systematic review and meta-analysis. Sex Transm Infect. 2013;89:251–6. 10.1136/sextrans-2012-050622 23024223

[12] Gayet-Ageron A, Ninet B, Toutous-Trellu L, Lautenschlager S, Furrer H, Piguet V, et al. Assessment of a real-time PCR test to diagnose syphilis from diverse biological samples. Sex Transm Infect. 2009;85:264–9. 10.1136/sti.2008.034314 19155240

[13] Shields M, Guy RJ, Jeoffreys NJ, Finlayson RJ, Donovan B. A longitudinal evaluation of *Treponema pallidum* PCR testing in early syphilis. BMC Infect Dis. 2012;12:353. 10.1186/1471-2334-12-353 23241398 PMC3541217

[14] Zhou C, Zhang X, Zhang W, Duan J, Zhao F. PCR detection for syphilis diagnosis: Status and prospects. J Clin Lab Anal. 2019;33:e22890. 10.1002/jcla.22890 30938474 PMC6595358

[15] Leslie DE, Azzato F, Karapanagiotidis T, Leydon J, Fyfe J. Development of a real-time PCR assay to detect *Treponema pallidum* in clinical specimens and assessment of the assay’s performance by comparison with serological testing. J Clin Microbiol. 2007;45:93–6. 10.1128/JCM.01578-06 17065262 PMC1828986

[16] Costa-Silva M, Coutinho D, Sobrinho-Simões J, Azevedo F, Lisboa C. Cross-sectional study of *Treponema pallidum* PCR in diagnosis of primary and secondary syphilis. Int J Dermatol. 2018;57:46–9. 10.1111/ijd.13823 29090453

[17] Theel ES, Katz SS, Pillay A. Molecular and direct detection tests for *Treponema pallidum* subspecies *pallidum*: a review of the literature, 1964–2017. Clin Infect Dis. 2020;71(Suppl 1):S4–12. 10.1093/cid/ciaa176 32578865 PMC7312206

[18] Towns JM, Leslie DE, Denham I, Wigan R, Azzato F, Williamson DA, et al. *Treponema pallidum* detection in lesion and non-lesion sites in men who have sex with men with early syphilis: a prospective, cross-sectional study. Lancet Infect Dis. 2021;21:1324–31. 10.1016/S1473-3099(20)30838-0 33894904

[19] Towns JM, Chow EPF, Wigan R, Fairley CK, Williamson D, Azzato F, et al. Anal and oral detection of *Treponema pallidum* in men who have sex with men with early syphilis infection. Sex Transm Infect. 2022;98:570–4. 10.1136/sextrans-2021-055370 35618414

[20] Nieuwenburg SA, Zondag HCA, Bruisten SM, Jongen VW, Schim van der Loeff MF, van Dam AP, et al. Detection of *Treponema pallidum* DNA during early syphilis stages in peripheral blood, oropharynx, ano-rectum and urine as a proxy for transmissibility. Clin Infect Dis. 2022;75:1054–62. 10.1093/cid/ciac056 35079776 PMC9522397

[21] Tantalo LC, Mendoza H, Katz DA, Sahi SK, Marra CM. Detection of *Treponema pallidum* DNA in oropharyngeal swabs and whole blood for syphilis diagnosis. Sex Transm Dis. 2021;48:915–8. 10.1097/OLQ.0000000000001476 34030158 PMC8595773

[22] Wang C, Hu Z, Zheng X, Ye M, Liao C, Shang M, et al. A new specimen for syphilis diagnosis: evidence by high loads of *Treponema pallidum* DNA in saliva. Clin Infect Dis. 2021;73:e3250–8. 10.1093/cid/ciaa1613 33099614 PMC8563222

[23] Yang CJ, Chang SY, Wu BR, Yang SP, Liu WC, Wu PY, et al. Unexpectedly high prevalence of *Treponema pallidum* infection in the oral cavity of human immunodeficiency virus-infected patients with early syphilis who had engaged in unprotected sex practices. Clin Microbiol Infect. 2015;21:787.e1–7. 10.1016/j.cmi.2015.04.018 25964151

[24] Bellhouse C, Walker S, Fairley CK, Chow EP, Bilardi JE. Getting the terminology right in sexual health research: the importance of accurately classifying fuck buddies among men who have sex with men. Sex Transm Infect. 2018;94:487–9. 10.1136/sextrans-2016-053000 28356437

[25] The Australasian Society for HIV, Viral Hepatitis and Sexual Health Medicine. Australian STI management guidelines for use in primary care. 2021 [cited 2023 Aug 1]. https://www.sti.guidelines.org.au

[26] Melbourne Sexual Health Centre. Testing guidelines. 2021 [cited 2023 Aug 1]. https://www.mshc.org.au/health-professionals/testing-guidelines

[27] Workowski KA, Bachmann LH, Chan PA, Johnston CM, Muzny CA, Park I, et al. Sexually transmitted infections treatment guidelines, 2021. MMWR Recomm Rep. 2021;70:1–187. 10.15585/mmwr.rr7004a1 34292926 PMC8344968

[28] Public Health Laboratory Network Australian Government Department of Health. Syphilis laboratory case definition, 2012 [cited 2023 Feb 20]. http://www.health.gov.au/ internet/main/publishing.nsf/Content/cda-phln-syphilis.htm

[29] Golden M, O’Donnell M, Lukehart S, Swenson P, Hovey P, Godornes C, et al. *Treponema pallidum* nucleic acid amplification testing to augment syphilis screening among men who have sex with men. J Clin Microbiol. 2019;57:e00572–19. 10.1128/JCM.00572-19 31189578 PMC6663910

[30] Mehta SD, Pradhan AK, Green SJ, Naqib A, Odoyo-June E, Gaydos CA, et al. Microbial diversity of genital ulcers of HSV-2 seropositive women. Sci Rep. 2017;7:15475. 10.1038/s41598-017-15554-8 29133803 PMC5684367

[31] Noguchi H, Tokumitsu T, Kuroki E, Minematsu E, Asada Y, Kuroda S, et al. Detection of *Treponema pallidum* by immunocytochemistry of cervical smear: A case report. Diagn Cytopathol. 2021;49:E443–6. 10.1002/dc.24849 34378872

[32] Grange PA, Gressier L, Dion PL, Farhi D, Benhaddou N, Gerhardt P, et al. Evaluation of a PCR test for detection of *treponema pallidum* in swabs and blood. J Clin Microbiol. 2012;50:546–52. 10.1128/JCM.00702-11 22219306 PMC3295187

[33] Getman D, Lin M, Barakat N, Skvoretz R, Godornes C, Swenson P, et al. Analytical performance characteristics of a new transcription-mediated amplification assay for *Treponema pallidum.* J Clin Microbiol. 2021;59:e0051121. 10.1128/JCM.00511-21 33980645 PMC8373238

